# Multi-omics landscape of alternative splicing in diffuse midline glioma reveals immune- and neural-driven subtypes with implications for spliceosome-targeted therapy

**DOI:** 10.3389/fimmu.2025.1587009

**Published:** 2025-04-16

**Authors:** Chaxian Liu, Yue Liu, Hao Lin, Chufan Zhang, Bilong Zhang, Haikun Song, Xiaomin Fan, Yi Lyu, Hui Yang, Ying Mao

**Affiliations:** ^1^ Department of Neurosurgery, Huashan Hospital, Fudan University, Shanghai, China; ^2^ Institute for Translational Brain Research, Shanghai Medical College, Fudan University, Shanghai, China; ^3^ National Center for Neurological Disorders, Huashan Hospital, Shanghai Medical College, Fudan University, Shanghai, China; ^4^ Shanghai Key Laboratory of Brain Function Restoration and Neural Regeneration, Shanghai Clinical Medical Center of Neurosurgery, Neurosurgical Institute of Fudan University, Huashan Hospital, Shanghai Medical College, Fudan University, Shanghai, China; ^5^ State Key Laboratory of Medical Neurobiology and MOE Frontiers Center for Brain Science and MOE Frontiers Center for Brain Science, Shanghai Medical College, Fudan University, Shanghai, China

**Keywords:** DMG, H3K27-altered, multi-omics, alternative splicing, metabolism, immune, posttranscriptional modifications, tumorigenesis

## Abstract

**Introduction:**

H3K27-altered diffuse midline glioma (DMG) is a highly aggressive glioma subtype, accounting for approximately 60% of pediatric high-grade gliomas, with a median survival of less than 12 months. Due to its predominant localization in the brainstem, conventional surgical resection is often unfeasible, underscoring the urgent need for alternative therapeutic strategies. While previous studies on DMG have primarily focused on regulatory mechanisms at the protein level, the role of alternative splicing in DMG remains largely unexplored. Given its potential impact on gene regulation and tumor progression, a comprehensive analysis of alternative splicing could provide novel insights into targeted or immune therapeutic strategies, complementing existing transcriptomic studies of DMG.

**Methods:**

To investigate the alternative splicing landscape of DMG, we performed transcriptome sequencing (RNA-seq) on patient-derived H3WT and H3K27-altered DMG cell lines, integrating these data with RNA-seq and single-cell transcriptomic (scRNA-seq) datasets from published sources. This comprehensive approach enabled us to delineate the alternative splicing landscape of H3K27-altered DMG and validate its distinct features at the cellular level.

**Results:**

Our multi-omics analysis revealed significant transcriptional alterations in H3K27-altered DMG compared to H3WT DMG, particularly in pathways related to neuro-regulation, metabolism, and immunity. Further in-depth analysis identified extensive alternative splicing changes in H3K27-altered DMG, predominantly associated with RNA modifications and key alterations in extracellular matrix and nucleotide metabolism. Integrating these findings, we characterized five RNA-associated proteins that enabled a binary classification of DMG into neural and immune subtypes, with each subtype exhibiting distinct prognostic and transcriptomic features. Notably, we identified *RALYL* as a potential key regulator in DMG progression.

**Discussion:**

Our findings indicate that H3K27-altered DMG exhibits significant alternative splicing alterations, which play crucial roles in tumorigenesis and progression. Additionally, our study identified an RNA-binding protein-based classification of DMG and characterized *RALYL* as a potential regulatory factor, highlighting its potential as a novel therapeutic target.

## 1 Introduction

Diffuse midline gliomas (DMG), particularly those with H3K27M alterations, are the most common high-grade gliomas in children (1). With a median survival of less than one year, DMG predominantly arises in midline structures and exhibits extensive invasiveness, rendering conventional surgical approaches largely ineffective (2–4). Current treatment strategies primarily rely on radiotherapy and chemotherapy, while emerging therapies, including CAR-T (5), tumor vaccines (6), and small-molecule drugs (7), are being actively explored. However, treatment responses vary significantly among patients. Recent multi-omics studies—including transcriptomics, epigenomics, proteomics, and metabolomics—have provided valuable insights into the internal heterogeneity and molecular characteristics of DMG, laying the foundation for potential targeted interventions (8–10).

Post-transcriptional regulation plays a crucial role in modulating gene expression at the mRNA level, encompassing processes such as conventional modifications, translation, and alternative splicing (AS) (11). In eukaryotes, mRNA undergoes intricate processing before translation, including the splicing, modification, or selective inclusion/exclusion of introns and exons—a process known as alternative splicing (12). Alternative splicing events generally include retained intron (RI), alternative 5’ splice site (A5SS), alternative 3’ splice site (A3SS), mutually exclusive exon (MXE), and skipped exon (SE) (13). This mechanism generates transcript diversity, contributing to protein structure and function variations, which may underlie intratumoral heterogeneity in tumors. Numerous studies have demonstrated that alternative splicing within tumors can significantly influence tumorigenesis, tumor progression, and immune evasion (14–16), including gliomas (17). However, as a distinct glioma subtype, DMG remains largely unexplored in this context, highlighting the need for further investigation.

In this study, we conducted an integrated analysis of H3WT and H3K27-altered DMG cell line samples, incorporating transcriptome sequencing (RNA-seq) data from patient tissue samples and single-cell transcriptome sequencing (scRNA-seq) data. This comprehensive approach enabled us to characterize the alternative splicing landscape of H3K27-altered DMG and identify distinct alternative splicing signatures. These events were predominantly associated with metabolic regulation, immune regulation, and RNA modification. By integrating RNA-binding protein (RBP) gene sets, we further demonstrated that H3K27-altered DMG could be dichotomized based on differentially expressed RBPs, with the two subtypes primarily linked to neuro-regulation and immune regulation, each representing distinct prognostic features. Notably, we identified *RALYL* as a key gene significantly correlated with poor prognosis in DMG, potentially contributing to tumor stemness and proliferation. In conclusion, our study provides the first comprehensive characterization of the alternative splicing landscape in DMG, unveils the intratumoral heterogeneity driven by alternative splicing, and highlights *RALYL* as a promising therapeutic target for DMG through alternative splicing-based mechanisms.

## 2 Results

### 2.1 H3WT and H3K27-altered DMG showed significant transcriptional landscape differences

**Figure 1:**
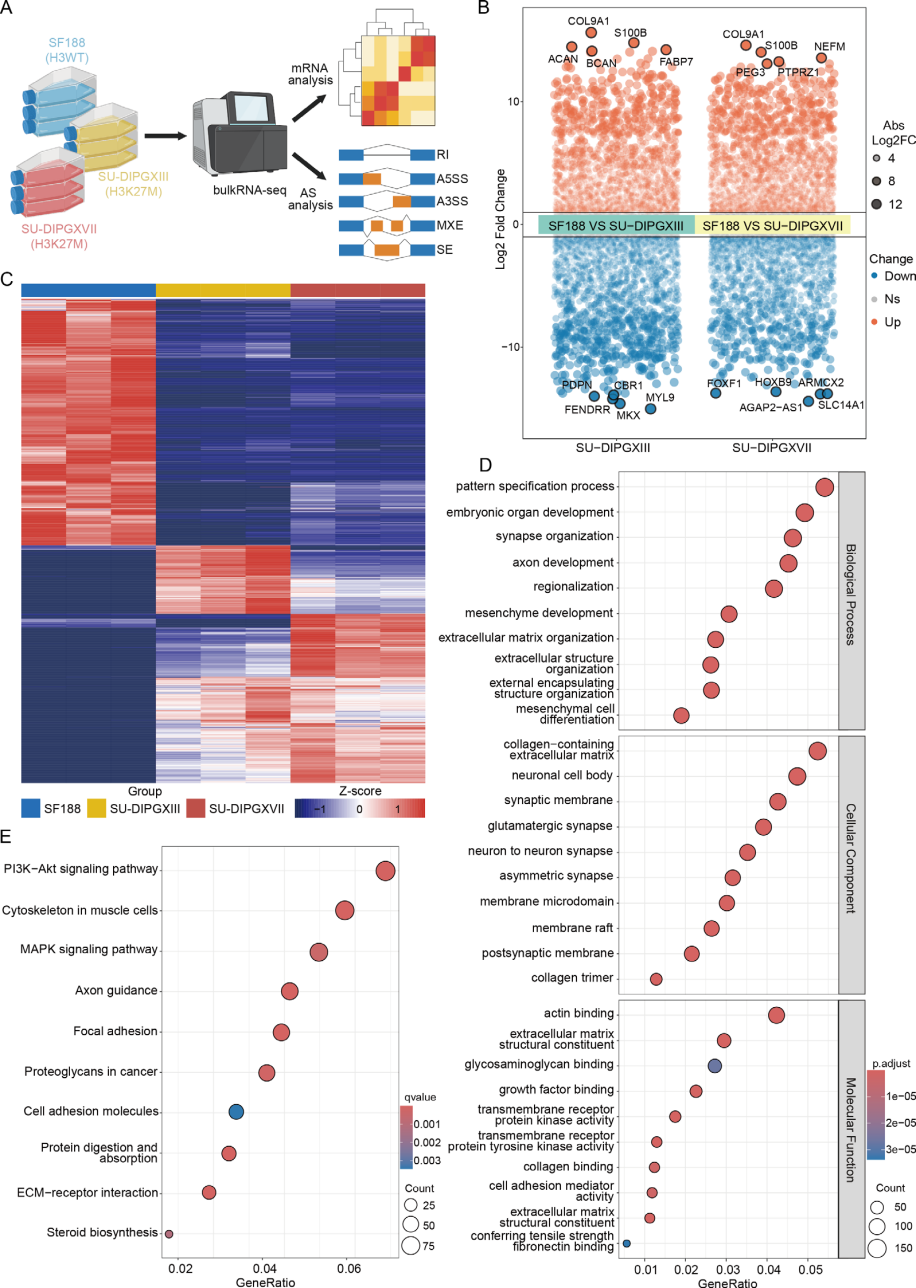
Differences in the transcriptional landscape between H3WT and H3K27-altered DMG. **(A)** The workflow of analysis of RNA-seq data. **(B)** A volcano plot of differentially expressed genes between SF188 and SU-DIPGXIII/SU-DIPGXVII and the five most significantly up-regulated/down-regulated genes are shown. **(C)** The heatmap of differentially expressed genes between SF188 and SU-DIPGXIII/SU-DIPGXVII. **(D)** GO enrichment results of upregulated differential genes shared between SU-DIPGXIII and SU-DIPGXVII. **(E)** KEGG enrichment results of upregulated differential genes shared between SU-DIPGXIII and SU-DIPGXVII.

To investigate transcriptomic alterations in H3K27-altered DMG, we performed RNA-seq by comparing H3WT (SF188) and H3K27M DIPG cell lines (SU-DIPGXIII and SU-DIPGXVII) (18) in three repeat samples (**Figure 1A**). Differential gene expression analysis was conducted on the raw data, and a volcano plot was generated to highlight key differentially expressed genes between SF188 and SU-DIPGXIII/SU-DIPGXVII (**Figure 1B** and **Supplementary Data Sheet 1**), with *COL9A1* and *S100B* showing significant upregulation (**Supplementary Figure 1**). A heatmap of these differentially expressed genes (**Figure 1C**) further demonstrated distinct gene expression patterns between H3WT and H3K27M DIPG cell lines. Subsequently, we selected genes that were consistently upregulated in H3K27M cell lines for Gene Ontology (GO) (**Figure 1D**) and KEGG pathway enrichment analysis (**Figure 1E**). The enrichment results revealed that H3K27-altered DMG tumor cells exhibited significant upregulation of neural-related pathways, including synapse organization, glutamatergic synapses, axon guidance, etc. Additionally, as a more stem-like and aggressive tumor type, H3K27M DIPG demonstrated enhanced mesenchyme development, extracellular matrix remodeling, and altered glucose and lipid metabolism, further supporting its highly invasive nature.

In conclusion, our preliminary RNA-seq analysis confirmed previously reported characteristics of H3K27-altered DMG, including pronounced neuro-synaptic features, strong tumor stemness, invasive properties, and enhanced glucose and lipid metabolism.

### 2.2 Differential alternative splicing exhibits a unique transcriptional landscape in DMG

**Figure 2:**
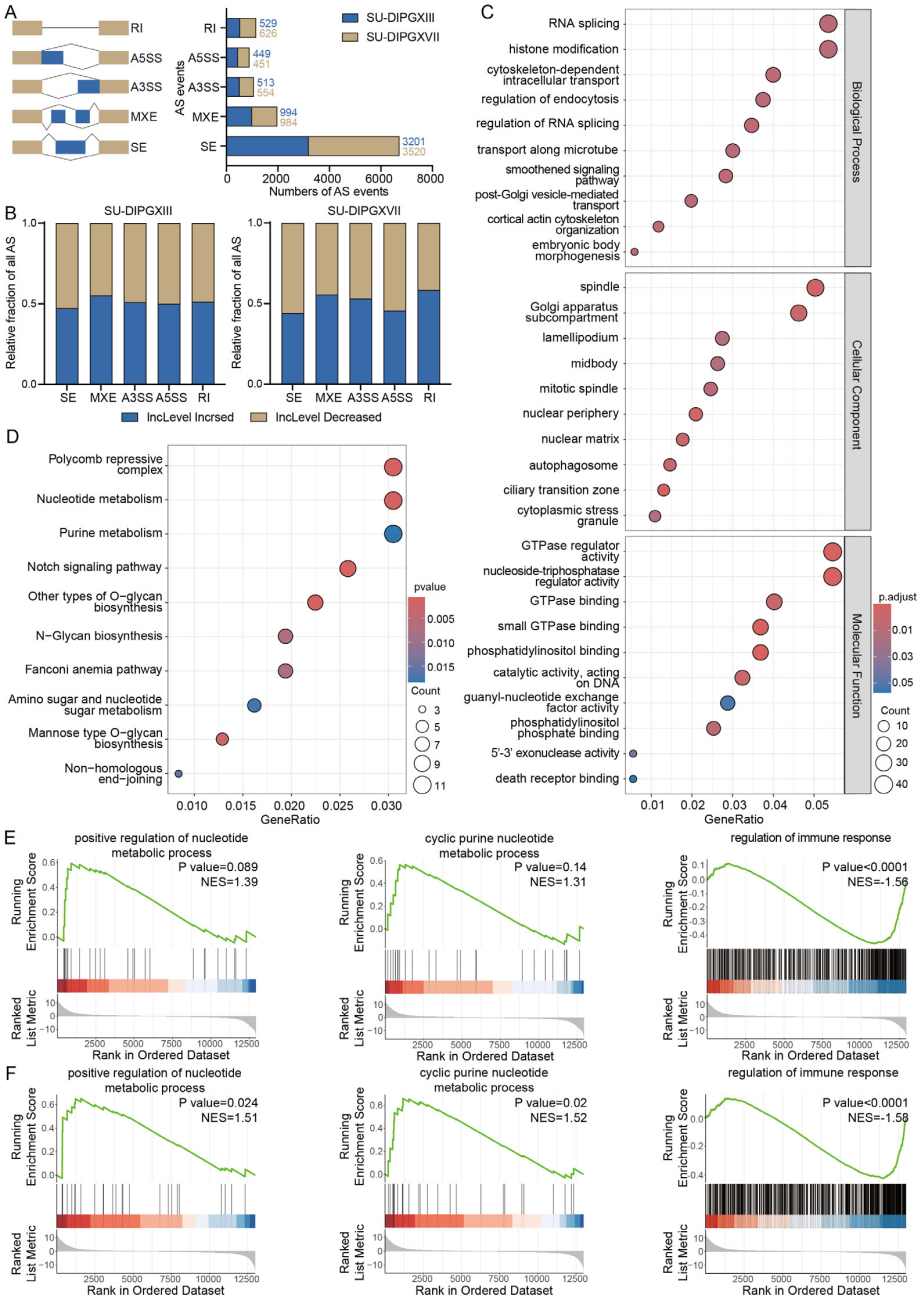
Differential analysis of alternative splicing in H3K27-altered DMG. **(A)** The overview of all differential alternative splicing events in SU-DIPGXIII and SU-DIPGXVII. **(B)** The overview of all differential alternative splicing events proportion in SU-DIPGXIII and SU-DIPGXVII. **(C)** GO enrichment results of upregulated differential alternative splicing genes shared between SU-DIPGXIII and SU-DIPGXVII. **(D)** KEGG enrichment results of upregulated differential alternative splicing genes shared between SU-DIPGXIII and SU-DIPGXVII. **(E)**The GSEA enrichment results of nucleotide metabolism-associated pathways and regulation of immune response in SU-DIPGXIII. **(F)** The GSEA enrichment results of nucleotide metabolism-associated pathways and regulation of immune response in SU-DIPGXVII.

We then utilized rMATS (19) to perform alternative splicing analysis on RNA-seq data from H3WT and H3K27M DIPG cell lines (**Supplementary Data Sheets 2** and **3**). Our results revealed many alternative splicing events between H3WT and H3K27M cells, with SE being the most prevalent event type (**Figure 2A**). Notably, the differential alternative splicing events between SU-DIPGXIII and SU-DIPGXVII remained broadly consistent (**Figure 2B**). To assess potential event preferences among these alternative splicing genes, we selected and integrated co-upregulated alternative splicing genes shared between SU-DIPGXIII and SU-DIPGXVII, followed by downstream GO and KEGG analyses (**Figures 2C, D**). Our findings indicate that the upregulated alternative splicing genes in H3K27M cells primarily involve RNA splicing, histone modification, nucleoplasmic transport, and nucleotide metabolism. Given that these pathways are involved in autophagy, apoptosis, nucleotide metabolism, and nucleotide translocation processes, immune components may also be potentially regulated. We conducted GSEA analysis on differentially expressed genes in SU-DIPGXIII and SU-DIPGXVII to further validate these findings. Compared to SF188, both SU-DIPGXIII (**Figure 2E**) and SU-DIPGXVII (**Figure 2F**) exhibited enrichment in nucleotide-related metabolic pathways and immune response, where nucleotide metabolism was upregulated, and immune response was downregulated in H3K27-altered DMG samples compared to H3WT samples, especially in SU-DIPGXVII.

In summary, alternative splicing events driven by H3K27M mutation are predominantly enriched in pathways related to RNA modification, nucleoplasmic transport, immune regulation, and nucleotide metabolism pathways. This suggests that alternative splicing plays a crucial role in sustaining the highly proliferative nature of H3K27-altered DMG. Targeting these splicing-regulated pathways may offer a potential strategy to disrupt tumor cell division and inhibit tumor progression with immune regulation.

### 2.3 Integrated transcriptome data reveal the metabolic-immune regulation of AS events

**Figure 3:**
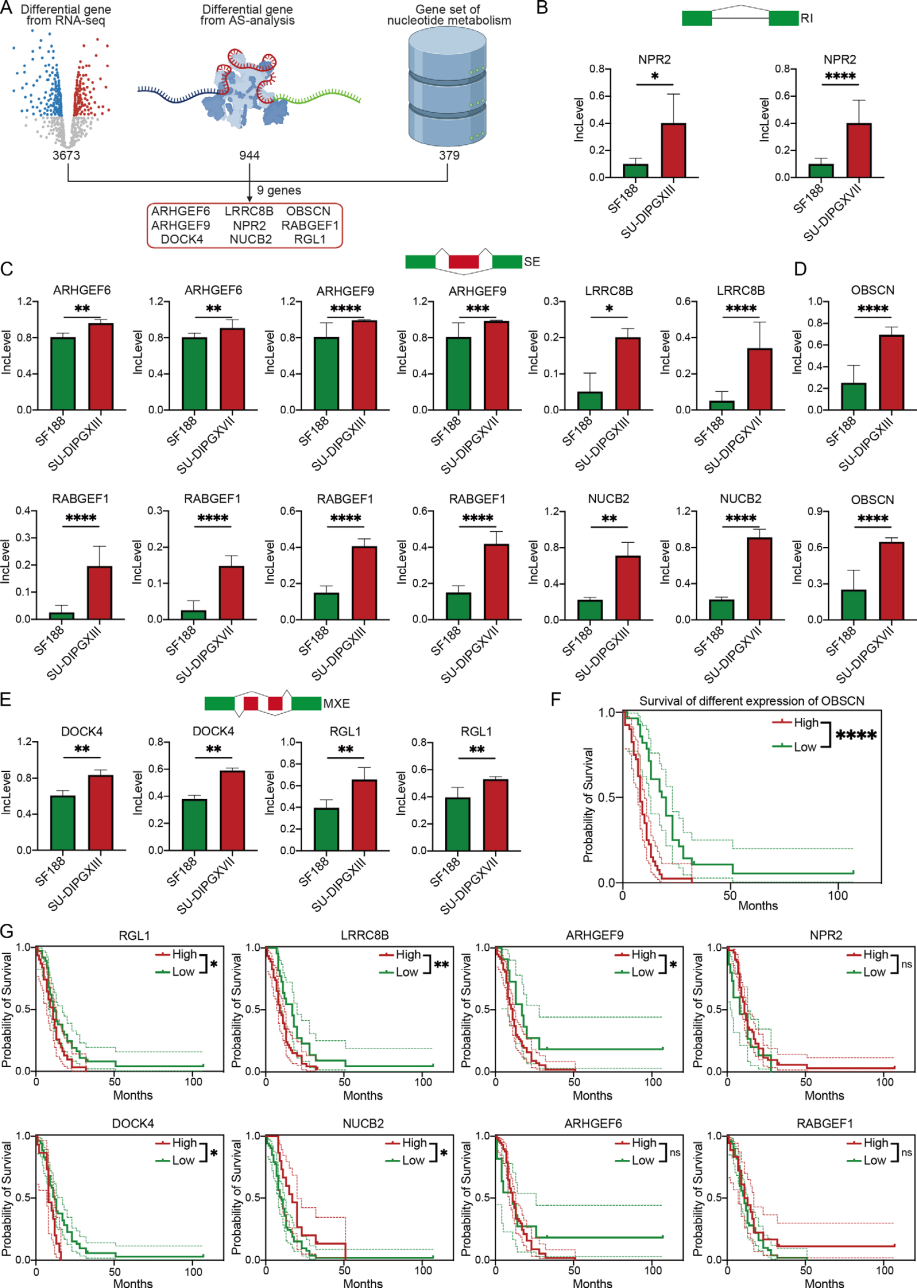
Integrated RNA-seq results revealed AS events of genes involved in nucleotide metabolism. **(A)** The workflow of analysis of integrating three data. **(B)** The Inclevel of unique genes between H3WT and H3K27M DIPG cell lines of RI. **(C, D)** The Inclevel of unique genes between H3WT and H3K27M DIPG cell lines of SE, and the RABGEF1 has two different SE event. **(E)** The Inclevel of unique genes between H3WT and H3K27M DIPG cell lines of MXE. **(F)** The survival analysis of different expressions of OBSCN. **(G)** The Kaplan–Meier survival analysis of different expressions of differential alternative splicing genes. For Inclevel, the Likelihood-ratio test was used for calculating the P value, considering P<0.05 to be significant, specifying *P<0.05, **P<0.01, ***P<0.001, ****P<0.0001, ns. P >0.05.

As the predominant pathways in AS events, to investigate the regulatory role of alternative splicing events in nucleotide metabolism in DMG, we integrated three datasets: 3,673 differentially expressed genes identified by RNA-seq between SU-DIPGXIII and SU-DIPGXVII, 944 upregulated differentially spliced genes, and 379 nucleotide metabolism-related genes derived from GSEA analysis (**Figure 3A**). This integrative approach identified nine key genes—*ARHGEF6*, *ARHGEF9*, *LRRC8B*, *OBSCN*, *NPR2*, *RABGEF1*, *DOCK4*, *NUCB2*, and *RGL1*—all of which exhibited distinct alternative splicing events and were consistently upregulated in both SU-DIPGXIII and SU-DIPGXVII (**Figures 3B–E**). Specifically, *NPR2* primarily underwent RI events, while *ARHGEF6*, *ARHGEF9*, *LRRC8B*, *NUCB2*, *OBSCN*, and *RABGEF1* were predominantly subject to SE events. MXE events were notably observed in *DOCK4* and *RGL1*. Additionally, *RABGEF1* exhibited two distinct SE events in SU-DIPGXIII and SU-DIPGXVII, whereas *OBSCN* displayed two unique SE events exclusively in SU-DIPGXIII.

**Figure 4:**
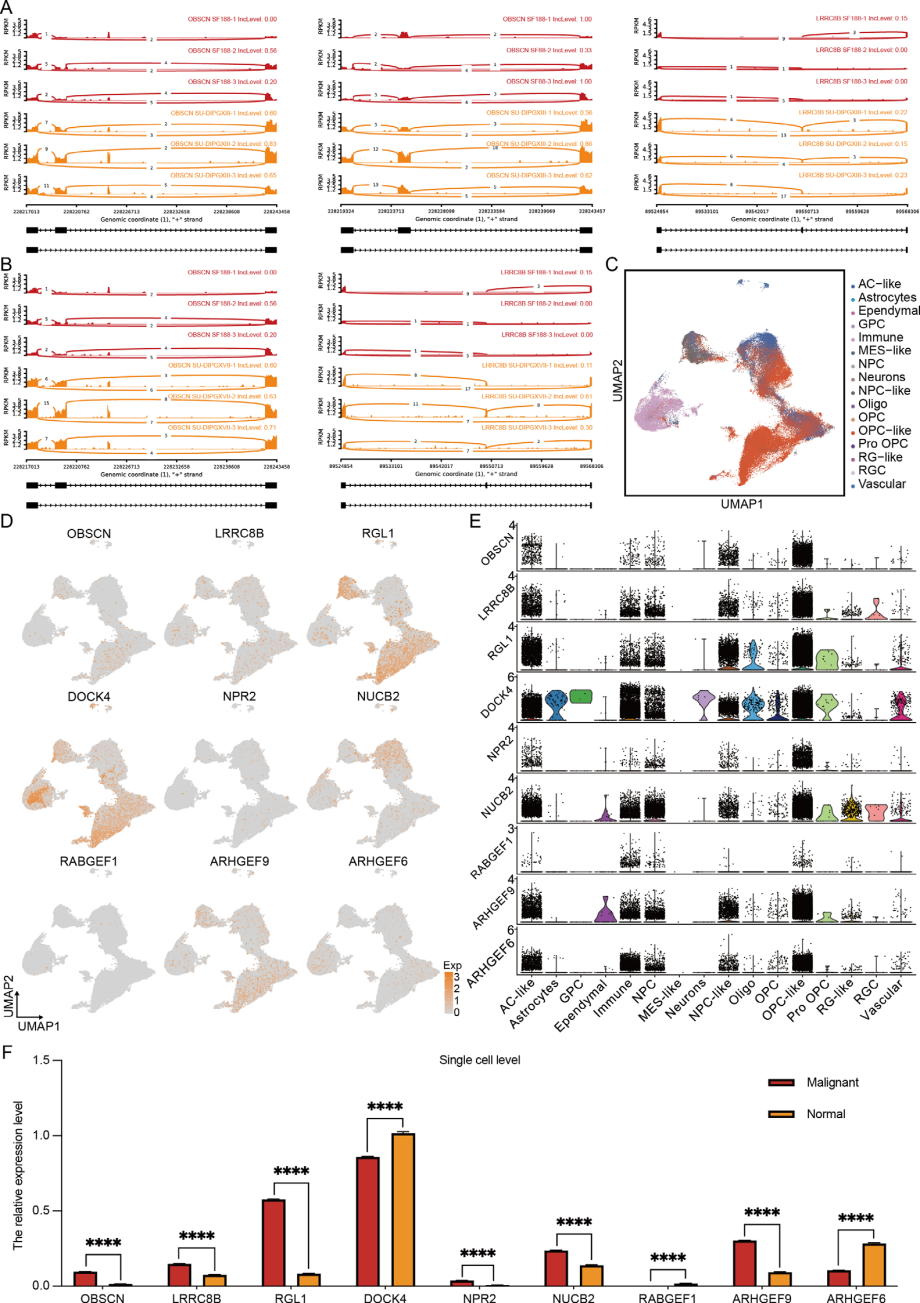
Multi-omics results reveal specific alternative splicing events in H3K27-altered DMG. **(A, B)** The overview of alternative splicing events in the genome. **(C)** The cluster of H3K27-altered DMG scRNA-seq overview. **(D)** The expression level of 9 alternative splicing genes in the single-cell levels. **(E)** The quantification of 9 alternative splicing genes in different cell types. **(F)** The quantification of 9 alternative splicing genes in malignant and normal clusters. The unpaired Student’s t test was used for P value calculation. ****P<0.0001.

To further refine our candidate genes, we leveraged the Pacific Pediatric Neuro-Oncology Consortium (PNOC) database to assess the prognostic relevance of the nine identified genes in DMG. Our analysis revealed that six genes—*OBSCN*, *LRRC8B*, *RGL1*, *ARHGEF9*, *DOCK4*, and *NUCB2*—were significantly negatively correlated with survival in H3K27-altered DMG patients (**Figures 3F, G**). Among them, *OBSCN* and *LRRC8B* exhibited the strongest association with poor prognosis. Additionally, we visualized the alternative splicing events of these two genes (**Figures 4A, B**), further highlighting their potential roles in DMG progression.

To further delineate the expression profiles of these candidate genes, we integrated the single-cell transcriptomic dataset of Jessa et al (20). We obtained a high-resolution single-cell dataset of H3K27M DMG comprising 76,112 cells (**Figure 4C**). Characterization (**Figure 4D**) and quantification (**Figure 4F**) of the nine genes revealed that *RGL1*, *DOCK4*, and *ARHGEF9* exhibited the highest expression levels in H3K27M DMG, followed by *OBSCN*, *LRRC8B*, and *NUCB2*. Notably, only *OBSCN*, *LRRC8B*, *RGL1*, *NUCB2*, and *ARHGEF9* showed significant upregulation in malignant cells. Further cellular subtype-level quantification (**Figure 4E**) demonstrated that *DOCK4* was predominantly expressed in immune components. In contrast, *OBSCN*, *LRRC8B*, *RGL1*, *ARHGEF9*, and *NUCB2* were enriched in OPC-like and AC-like cells—the primary cellular subsets of DMG (21). The accumulation of these upregulated alternative splicing events within these key cell populations suggests their potential role in driving DMG progression and invasiveness, especially *RGL1* and *ARHGEF9*.

To validate the role of *RGL1* and *ARHGEF9* in H3K27M DMG, and to assess its functional impact, we performed *RGL1* and *ARHGEF9* knockdown (**Supplementary Figures 2A, B**). Cell proliferation assays (**Supplementary Figures 2C, D**) demonstrated that *RGL1* and *ARHGEF9* knockdown significantly inhibited the proliferation of H3K27M DIPG cells. Additionally, limiting dilution analysis (**Supplementary Figures 2E, F**) and *in vitro* sphere-forming assays (**Supplementary Figures 2G–J**) showed that *RGL1* and *ARHGEF9* knockdown markedly impaired the stemness maintenance of mutant cell lines. These findings suggest that *RGL1* and *ARHGEF9* are essential for proliferation and stemness maintenance in H3K27-altered DMG tumor cells, highlighting *RGL1* and *ARHGEF9* as potential therapeutic targets for future DMG treatment.

In conclusion, our integrated multi-omics analysis identified *OBSCN*, *LRRC8B*, *RGL1*, *NUCB2*, and *ARHGEF9* as potential alternative splicing events driving malignant cell progression. At the same time, *DOCK4* emerged as a key alternative splicing event associated with the immune microenvironment that may be involved in bridging the association between immunity and metabolism. Among them, *RGL1* and *ARHGEF9* stand out as a particularly noteworthy candidate, further *in vitro* experiments verified the effects of both on DMG proliferation and dryness.

### 2.4 Multi-omics analysis revealed neural-immune subtype by alternative splicing modifiers

**Figure 5:**
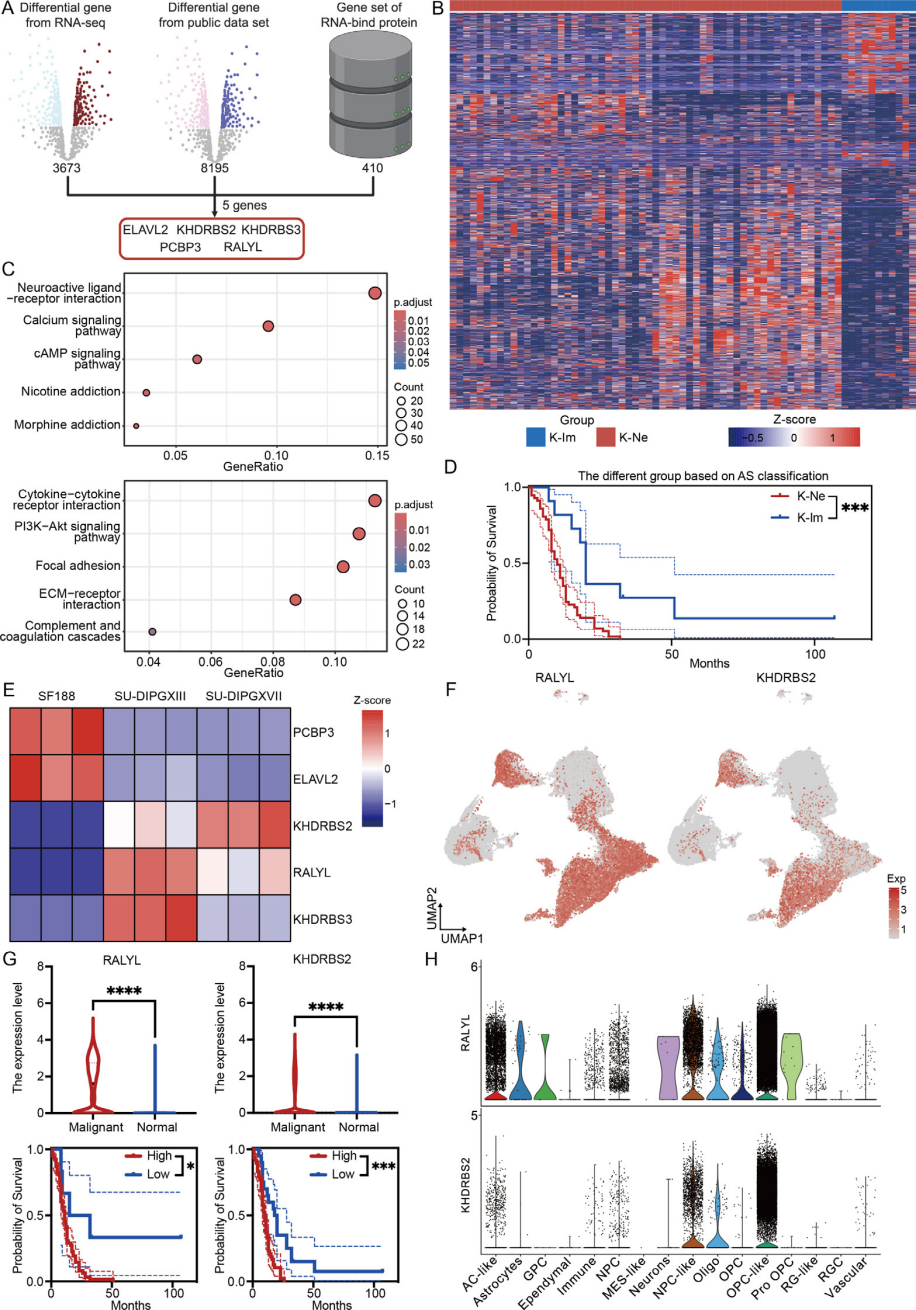
*RALYL* is the key alternative splicing modifier in H3K27-altered DMG. **(A)** The workflow of analysis integrates three data sets for RBP genes. **(B)** The heatmap of different subtypes depends on different expression levels of the modifier gene set. **(C)** KEGG enrichment results of upregulated differential genes in K-Ne (upper) and K-Im (bottom). **(D)** The Kaplan–Meier survival analysis of different subtypes between K-Ne and K-Im. **(E)** The heatmap of modifier genes based on cell line RNA-seq data. **(F)** The expression level of *RALYL* (left) and *KHDRBS2* (right) in the single-cell levels. **(G)** The quantification of the expression level of *RALYL* (upper left) and *KHDRBS2* (upper right) in malignant and normal clusters with the Kaplan–Meier survival analysis of different expressions of *RALYL* (bottom left) and *KHDRBS2* (bottom right). **(H)** The expression level of RALYL (upper) and KHDRBS2 (bottom) in different celltype in single-cell level. *P<0.05, ***P<0.001, ****P<0.0001.

Given that alternative splicing is regulated by interactions between RBPs and RNA, we sought to identify key RBP-associated gene sets involved in DMG pathogenesis. To achieve this, we integrated our RNA-seq differential gene dataset, the PNOC sample differential gene dataset, and RBP-related genes (**Supplementary Data Sheet 4**). This analysis identified five significantly dysregulated RBPs: *ELAVL2*, *KHDRBS2*, *KHDRBS3*, *PCBP3*, and *RALYL* (**Figure 5A**). Notably, hierarchical clustering based on these five genes stratified DMG samples into two distinct subgroups (**Figure 5B**). The first subgroup, characterized by high RBP expression, exhibited an upregulation of neural-related genes (**Figure 5C**, upper). In contrast, the second subgroup, with low RBP expression, showed enrichment in cytokine and immune-related pathways (**Figure 5C**, bottom). Based on these expression patterns, we classified DMG into two novel subtypes: K-Ne (neural-associated) and K-Im (immune-associated). Survival analysis revealed a significant prognostic distinction between these subtypes, with the K-Ne group exhibiting poorer outcomes. This classification framework provides a novel perspective on DMG heterogeneity and holds potential clinical relevance for stratifying patients, guiding therapeutic strategies, and revealing and predicting the strong effects of alternative splicing events on the immune microenvironment of DMG.

Furthermore, as the above-mentioned RNA-seq data showed that *COL9A1* and *S100B* were similarly highly expressed in SU-DIPGXIII and SU-DIPGXVII, we further examined whether these alternative splicing events were associated with these two genes. Using the expression matrix of PNOC, we performed correlation analysis. The results showed a weak correlation between *COL9A1* and these AS genes (**Supplementary Figure 3**), except for *ARHGEF9*, which showed a positive correlation. For RBP genes, *ELAVL2*, *KHDRBS3*, and *RALYL* showed a positive correlation. For *S100B* (**Supplementary Figure 4**), the correlation between *S100B* and AS genes was also weak. Except for *ARHGEF6*, *OBSCN* and *RABGEF1* showed a certain negative correlation. However, for RBP genes, *S100B* showed weaker associations with these genes. These results suggest that the correlation between highly expressed gene profiles and alternative splicing events in RNA-seq may not necessarily be consistent and may require further consideration of the physiological or pathological implications.

To further characterize these RBPs, we analyzed their expression in a cellular-level RNA-seq dataset (**Figure 5E**). Our findings revealed that *KHDRBS2* and *RALYL* were consistently upregulated in H3K27-altered samples compared to H3WT samples, a trend also observed at the single-cell level (**Figure 5F**). Further quantitative analysis demonstrated that both *RALYL* and *KHDRBS2* exhibited high expression levels in malignant cells and were significantly associated with worse prognoses in DMG patients (**Figure 5G**). However, when analyzing expression across distinct cellular subpopulations, we found that *RALYL* displayed higher expression in malignant fractions, particularly in AC-like, NPC-like, and OPC-like tumor cells, while being minimally expressed in normal cells (**Figure 5H**). This suggests that *RALYL* may serve as a tumor-specific RNA-modifying protein in DMG, potentially playing a critical role in disease progression.

In conclusion, by integrating and analyzing multi-omics data, we identified a classification system that divides DMG into two distinct subgroups based on the expression levels of five key RBP modifiers. These subgroups exhibit distinct neurological and immune-related characteristics and serve as prognostic indicators for patient outcomes. Further investigation revealed that *RALYL*, a modifier expressed explicitly in malignant cells, may be crucial in driving DMG progression and regulating alternative splicing events, offering a potential therapeutic target for this aggressive glioma subtype.

### 2.5 Targeting *RALYL* can inhibit tumor cell progression

**Figure 6:**
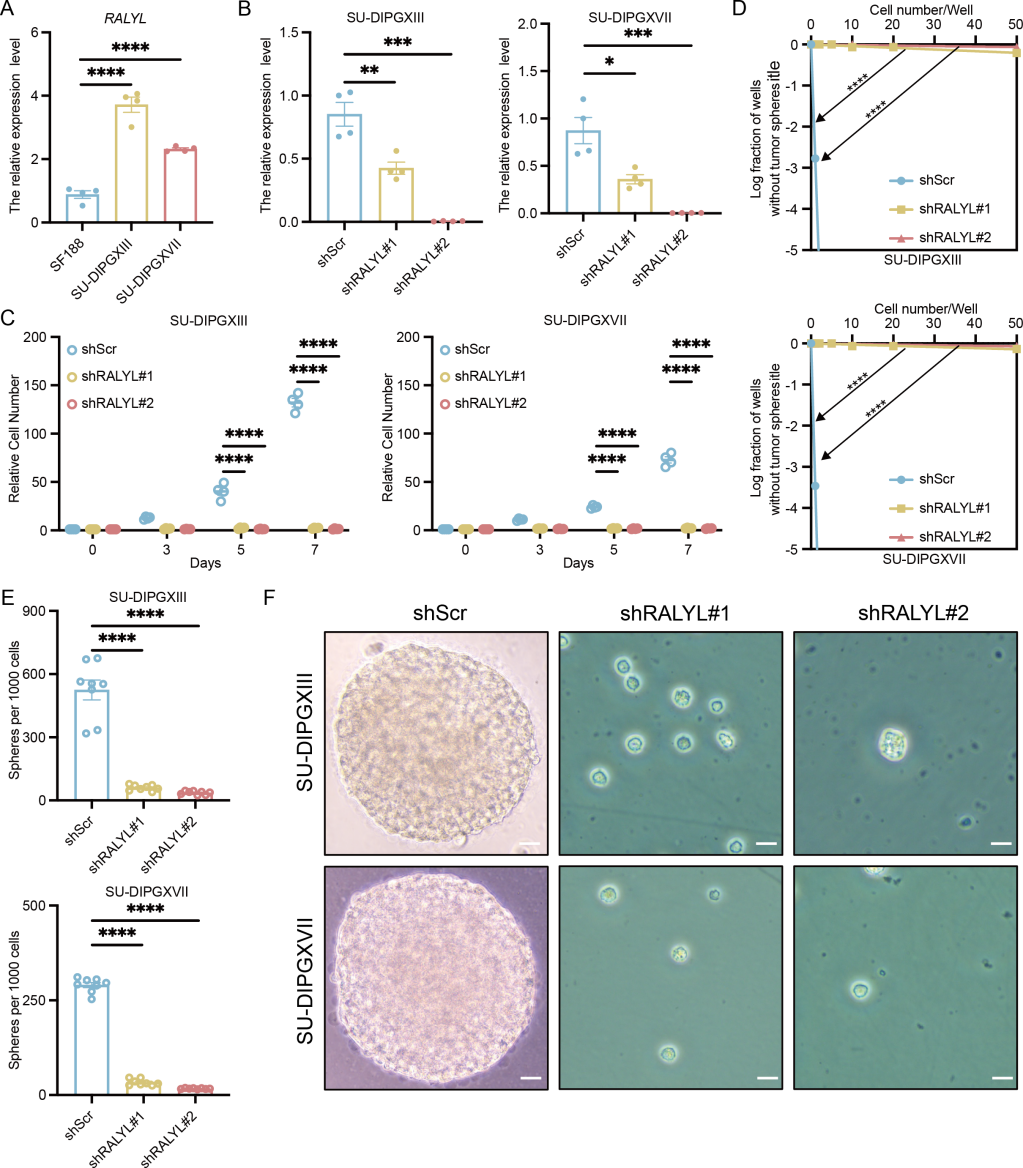
*RALYL* targeting significantly inhibited tumor cell growth and stemness. **(A)** The quantification of *RALYL* expression levels between SF188 and SU-DIPGXIII/SU-DIPGXVII. **(B)** The quantification of *RALYL* knockdown efficiency in SU-DIPGXIII (left) and SU-DIPGXVII (right). **(C)** The proliferation assay on cell number shows that *RALYL* knockdown impairs the proliferation of SU-DIPGXIII (left) and SU-DIPGXVII (right). **(D)** The limiting dilution assays in SU-DIPGXIII (upper) and SU-DIPGXVII (bottom) cell lines with *RALYL* knockdown. **(E, F)** The result of tumorsphere formation (scale bar = 20 μm) *in vitro* shows impairing self-renewal of *RALYL* knockdown in SU-DIPGXIII (upper) and SU-DIPGXVII (bottom) *P<0.05, **P<0.01, ***P<0.001, ****P<0.0001.

To validate the role of *RALYL* in H3K27M DMG, we first compared its expression levels in H3WT and H3K27M DIPG cell lines (**Figure 6A**), confirming that *RALYL* is significantly upregulated in H3K27M DIPG. To assess its functional impact, we performed *RALYL* knockdown (**Figure 6B**). Cell proliferation assays (**Figure 6C**) demonstrated that *RALYL* knockdown significantly inhibited the proliferation of H3K27M DIPG cells. Additionally, limiting dilution analysis (**Figure 6D**) and *in vitro* sphere-forming assays (**Figures 6E, F**) showed that *RALYL* knockdown markedly impaired the stemness maintenance of mutant cell lines. These findings suggest that *RALYL* is essential for proliferation and stemness maintenance in H3K27-altered DMG tumor cells, highlighting *RALYL* as a potential therapeutic target for future DMG treatment.

To further investigate the impact of *RALYL* on AS events in DMG, we selected the five AS-related genes highly expressed in malignant cells for *in vitro* validation. qRT-PCR results revealed that these AS genes were significantly upregulated following *RALYL* knockdown (**Supplementary Figure 5**). At first glance, this result seems contradictory, as both *RALYL* and these splicing-related genes are associated with malignancy. However, this finding is reasonable given that our study suggests these two factors operate relatively independently. Furthermore, considering the complex regulatory mechanisms in tumor cells and the multifaceted regulation of alternative splicing by multiple RBPs, this result highlights the intricate nature of splicing networks in H3K27M DMG. These findings underscore the need for a comprehensive approach when developing therapies targeting alternative splicing in DMG.

## 3 Discussion

As the most aggressive subtype of pediatric high-grade glioma, DMG—particularly H3K27-altered DMG is highly malignant and more prevalent. Due to its brainstem localization, conventional surgical interventions are not viable, making non-surgical treatments the primary therapeutic approach. However, the significant intratumoral heterogeneity of DMG results in highly variable treatment responses, underscoring the need to explore its molecular heterogeneity. In this study, we comprehensively characterized the alternative splicing landscape of H3K27-altered DMG for the first time, revealing that alternative splicing events and their associated regulatory proteins may serve as prognostic markers and potential therapeutic targets for DMG.

Our results revealed extensive alternative splicing events in H3K27-altered DMG compared to H3WT, with SE being the most prevalent. These events were primarily associated with RNA modification, nucleoplasmic translocation, immune regulation, and nucleotide metabolism, suggesting that alternative splicing may play a crucial in promoting DMG proliferation and invasion. Further analysis highlighted that upregulated alternative splicing genes were particularly enriched in nucleotide metabolism pathways. Through an integrated screening approach, we identified six key alternative splicing events (*OBSCN*, *LRRC8B*, *RGL1*, *DOCK4*, *NUCB2*, and *ARHGEF9*), with *RGL1* emerging as the most prominent. As a guanine nucleotide exchange factor and as an RAS effector, RGL1 may contribute to tumorigenesis through epigenetic regulation or induction of KRAS mutations (22, 23). Given the role of purine metabolism in drug resistance, targeting *RGL1* may represent a novel therapeutic strategy for DMG (24). On the other hand, another key splicing event, *DOCK4*, is highly expressed in immune cells within the tumor microenvironment. Previous studies have linked *DOCK4* to immune cell infiltration in tumors (25, 26), suggesting that its alternative splicing events in DMG may serve as a bridge between tumor metabolism and tumor immunity. Targeting *DOCK4* could be a potential strategy to regulate anti-tumor metabolism and immunity simultaneously.

Additionally, we found that H3K27-altered DMG could be further stratified based on the differential expression of five RBPs. This classification identified two distinct subtypes of DMG: one with strong neurological features and another with predominant immune-related characteristics. Transcriptome analysis of clinical samples validated this classification, revealing that the neurological subtype was associated with the worst prognosis, consistent with DMG’s known neurobiological characteristics. In contrast, the immune subtype correlated with better survival outcomes (8, 27, 28). This alternative splicing-based classification provides a valuable transcriptomic framework for understanding DMG heterogeneity and may guide future therapeutic strategies.

Among these RNA-binding protein modifiers, *RALYL* stood out as a gene specifically expressed in the malignant components of DMG. *RALYL*, a heterogeneous ribonucleoprotein (hnRNP) family member, plays a crucial role in RNA post-transcriptional modification (29). Previous studies have shown that *RALYL* promotes the stemness maintenance of cancer stem cells (30, 31), and our analysis further revealed its association with poor prognosis in DMG. Functional validation experiments demonstrated that *RALYL* knockdown in H3K27M DIPG cells significantly impaired both cell proliferation and stemness, suggesting that targeting *RALYL* could be a promising therapeutic strategy for DMG. Furthermore, given its selective expression in malignant tumor components, *RALYL*-based therapeutic approaches—such as targeted therapy, tumor vaccines, or CAR-T cell therapy—could offer potential clinical applications (32). Therefore, the RBP-based classification of DMG, combined with *RALYL*’s functional role, may provide new insights into the profound molecular heterogeneity of DMG. However, considering the complex and possibly independent variable splicing regulatory network within DMG, further studies are needed to validate these findings and explore their translational potential.

Taken together, H3K27-altered DMG exhibits a unique post-transcriptional modification landscape characterized by distinct alternative splicing events and intratumoral heterogeneity in RBP gene expression. Our study delineates the alternative splicing landscape of H3K27-altered DMG and defines a novel prognostic profile based on RBP gene analysis. Notably, our findings suggest that *RALYL* may serve as a unique therapeutic target, warranting further investigation.

## 4 Materials and methods

### 4.1 Patient-derived DMG cell and cell line culture

SF188 (H3WT), SU-DIPGXIII (H3.3K27M), and SU-DIPGXVII (H3.3K27M) were provided by Dr. Yujie Tang from Shanghai Jiao Tong University and in brief, SU-DIPGXIII was obtained from a biopsy of a 2-year-old female patient with DMG, while SU-DIPGXVII was obtained from a biopsy of an 8-year-old male with DMG (18). HEK293T (from ATCC) and SF188 were cultured in DMEM with 10% FBS and 1x penicillin/streptomycin. SU-DIPGXIII and SU-DIPGXVII were grown as previously described (18, 33) in tumor neurospheres in tumor stem cell medium, including DMEM/F12 (Thermo, 11330032), Neurobasal-A Medium (Thermo, 10888022), B27 (Thermo, 12587010), human PDGF-AA and PDGF-BB (20 ng/ml each, Shenandoah Biotech), human EGF and bFGF (20 ng/mL each, Shenandoah Biotech) and heparin (10 ng/ml, Stemcell).

### 4.2 Lentivirus preparation and infection

Two shRNAs targeting human RALYL (target sequence: 5’-CGGCAATCTAAATACGGCAAT-3’ and 5’-TGTTCCGTTCACAAAGGTTAT-3’), RGL1 (target sequence: 5’-CCTGTTTACAACCAACAGAAT-3’ and 5’-CGCATCAGTGTGGAAGACAAT-3’), ARHGEF9 (target sequence: 5’-GAGGAGATACATCTGTTCTTT-3’ and 5’-CGAGCAACTGAAGGTAATCTT-3’), and a non-targeting control shRNA (shScr, 5’-CCTAAGGTTAAGTCGCCCTCG-3’) were cloned into the PLKO.1. Lentivirus, psPAX2 (Addgene, #12259) and pMD2.G (Addgene, #12259) with PEI (Yeasen, #40815ES) co-transfected into HEK293T to produce lentiviral particles. Viruses were collected after transfection and precipitated by PEG8000 (Sigma, #89510). For lentiviral infection, cells were dissociated into single cells and incubated with concentrated viral particles for 24 h, followed by 0.5 μg/ml puromycin to select infected cells to obtain stable cells. Stable cells were detected by RT-qPCR.

### 4.3 qRT-PCR

Total cellular RNA was isolated using RNA purification kit (EZB, #B0004DP). And reverse transcribed using 4X EZscript Reverse Transcription Mix II (EZB, #RT2GQ). qRT-PCR was performed on a QuantStudio 6 Flex Real-Time PCR System (Thermo Fisher Scientific) using 2X Color SYBR Green qPCR Master Mix (EZB, #A0012-R2). The qPCR primer pairs in this study were in **Supplementary Data Sheet 5**.

### 4.4 *In vitro* limiting dilution assay

The limiting dilution assay was performed according to our previously reported protocol (34). The presence and number of tumor neurospheres per well were recorded 7 days after planting. Extreme limiting dilution analysis was performed at http://bioinf.wehi.edu.au/software/elda (35, 36).

### 4.5 Cell proliferation and neurosphere formation assay

Cell proliferation assay was performed by planting DMG cells at 10,000 cells/well in 6-well plates, with four repeats per group. Cell counts were performed on days 3, 5, and 7. Neurosphere formation was performed by planting DMG cells at 5,000 cells/well in 24-well plates, with eight repeats per group. The presence and number of tumor spheres in each well were recorded 7 days after planting.

### 4.6 RNA-seq data analysis

Total RNA was purified from SU-DIPGXIII, SU-DIPGXVII, and SF188 cells using TRIzol reagent (Invitrogen, #15596018CN) according to the manufacturer’s protocol for the subsequent library preparation. The RNA-seq library was loaded on an Illumina NovaSeq 6000 instrument for sequencing using a 2×150 paired-end (PE) configuration according to the manufacturer’s instructions. The mapped reads were aligned to the hg38 reference genome by STAR (v2.7.11b) for further analysis. For differential expression analysis, R package limma (v3.56.2) and DESeq2 (v1.40.2) were used to analyze the differences in DMG and the differential genes in P < 0.05 and |LogFC| > 1.2. For KEGG and GO enrichment, clusterProfiler (v4.8.3) was used for enrichment analysis, and ggplot2 (v3.5.1) for plotting. For the alternative splicing analysis, we used the rMATS (v4.3.0) and followed the protocol of Xing et al (37). For survival analysis, survival (v3.5-7) and survminer (v0.4.9) were used. The classification based on RBP genes used R package GSVA (v1.50.5) to calculate the gene set score for the quantification of expression levels also used in AS gene sets.

### 4.7 scRNA-seq data analysis

For the scRNA-seq, we followed the analysis procedure provided by Jessa et al. (20). In brief, we downloaded their dataset and then followed the code provided in the article for stepwise analysis to obtain harmony (v1.2.0) integration data with their original annotation results. Then, we identified cell subtypes by gene-set scoring using R package AUCell (v1.22.0) following the typing of the gene set results of Neftel et al. (38) to define the malignant cells. Plots were generated by Seurat (v5.1.0).

### 4.8 Quantitative and statistical analysis

R (v4.3.1) and python (v3.11.6) were used for all bioinformatics analyses. Prism software (version 10) was also used. Survival curves were statistically analyzed using the Log-rank test and plotted using Kaplan-Meier curves. For Inclevel, the Likelihood-ratio test was used to calculate the P value, followed by rMATS. For the rest of the data, unpaired, two-sided, Student’s t tests was used, and considered P<0.05 to be significant, specifying *P<0.05, **P<0.01, ***P<0.001, ****P<0.0001, ns. P >0.05.

## Data Availability

The data presented in the study are deposited in the OSF repository, accession link https://osf.io/dhpza/files/osfstorage?view_only=30eaf80bae964e40af7a8325d3db2095.

## Supplementary material

Supplementary Figure 1 The verification of every three upregulation or downregulation differential expression genes using qRT-PCR from the differential expression genes. **(A)** The verification of upregulation expression genes using qRT-PCR in SU-DIPGXIII and SU-DIPGXVII. **(B)** The verification of downregulation expression genes using qRT-PCR in SU-DIPGXIII. **(C)** The verification of downregulation expression genes using qRT-PCR in SU-DIPGXVII. *P<0.05, **P<0.01, ***P<0.001, ****P<0.0001.

Supplementary Figure 2
*RGL1* and *ARHGEF9* targeting significantly inhibited tumor cell growth and stemness. **(A)** The quantification of *RGL1* knockdown efficiency in SU-DIPGXIII (left) and SU-DIPGXVII (right). **(B)** The quantification of *ARHGEF9* knockdown efficiency in SU-DIPGXIII (left) and SU-DIPGXVII (right). **(C)** The proliferation assay on cell number shows that *RGL1* knockdown impairs the proliferation of SU-DIPGXIII (left) and SU-DIPGXVII (right). **(D)** The proliferation assay on cell number shows that *ARHGEF9* knockdown impairs the proliferation of SU-DIPGXIII (left) and SU-DIPGXVII (right). **(E)** The limiting dilution assays in SU-DIPGXIII (left) and SU-DIPGXVII (right) cell lines with *RGL1* knockdown. **(F)** The limiting dilution assays in SU-DIPGXIII (left) and SU-DIPGXVII (right) cell lines with *ARHGEF9* knockdown. **(G)** The quantitative result of tumorsphere formation *in vitro* of *RGL1* knockdown in SU-DIPGXIII (left) and SU-DIPGXVII (right). **(H)** The quantitative result of tumorsphere formation *in vitro* of *ARHGEF9* knockdown in SU-DIPGXIII (left) and SU-DIPGXVII (right). **(I)** The result of tumorsphere formation *in vitro* shows impairing self-renewal of *RGL1* knockdown in SU-DIPGXIII (left) and SU-DIPGXVII (right). **(J)** The result of tumorsphere formation (scale bar = 20 μm) *in vitro* shows impairing self-renewal of *ARHGEF9* knockdown in SU-DIPGXIII (left) and SU-DIPGXVII (right). ****P<0.0001.

Supplementary Figure 3 The correlation analysis between the expression of *COL9A1* and AS genes and RBP genes in DMG. **(A)** The correlation analysis between the expression of *COL9A1* and AS genes set in DMG. **(B)** The correlation analysis between the expression of *COL9A1* and each AS gene in DMG. **(C)** The correlation analysis between the expression of *COL9A1* and RBP genes set in DMG. **(D)** The correlation analysis between the expression of *COL9A1* and each RBP gene in DMG.

Supplementary Figure 4 The correlation analysis between the expression of *S100B* and AS genes and RBP genes in DMG. **(A)** The correlation analysis between the expression of *S100B* and AS genes set in DMG. **(B)** The correlation analysis between the expression of *S100B* and each AS gene in DMG. **(C)** The correlation analysis between the expression of *S100B* and RBP genes set in DMG. **(D)** The correlation analysis between the expression of *S100B* and each RBP gene in DMG.

Supplementary Figure 5 The qRT-PCR validation of five AS genes in H3K27M DMG cell after *RALYL* knockdown. **(A)** The qRT-PCR validation of five AS genes in SU-DIPGXIII cell after *RALYL* knockdown. **(B)** The qRT-PCR validation of five AS genes in SU-DIPGXVII cell after *RALYL* knockdown. *P<0.05, ***P<0.001, ****P<0.0001, ns. P >0.05.

Supplementary Data Sheet 1 The differential expression genes among the DMG cell lines by RNA-seq.

Supplementary Data Sheet 2 All events of SF188 and SU-DIPGXIII differential alternative splicing.

Supplementary Data Sheet 3 All events of SF188 and SU-DIPGXVII differential alternative splicing.

Supplementary Data Sheet 4 RNA binding protein-related gene set.

Supplementary Data Sheet 5 The qRT-PCR primer list.
