# Supplementary figures and images for: Multi-omics landscape of alternative splicing in diffuse midline glioma reveals immune- and neural-driven subtypes with implications for spliceosome-targeted therapy
Source: Front Immunol. 2025 Apr 16;16:1587009. doi: 10.3389/fimmu.2025.1587009 (PMC12040961; doi:10.3389/fimmu.2025.1587009)

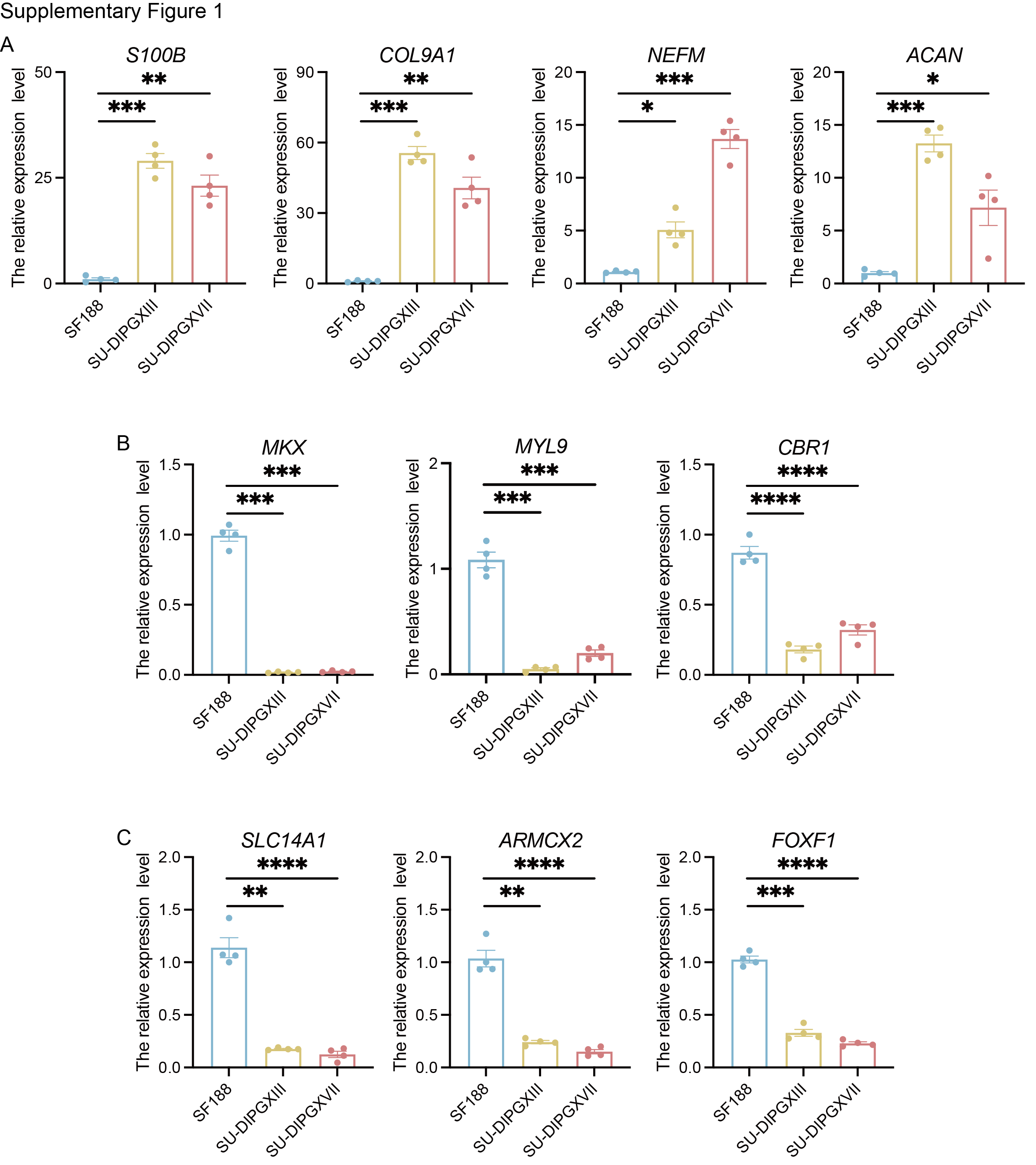

Supplement: Supplementary Figure 1 — The verification of every three upregulation or downregulation differential expression genes using qRT-PCR from the differential expression genes. (A) The verification of upregulation expression genes using qRT-PCR in SU-DIPGXIII and SU-DIPGXVII. (B) The verification of downregulation expression genes using qRT-PCR in SU-DIPGXIII. (C) The verification of downregulation expression genes using qRT-PCR in SU-DIPGXVII. *P<0.05, **P<0.01, ***P<0.001, ****P<0.0001. [file Image1.tif]

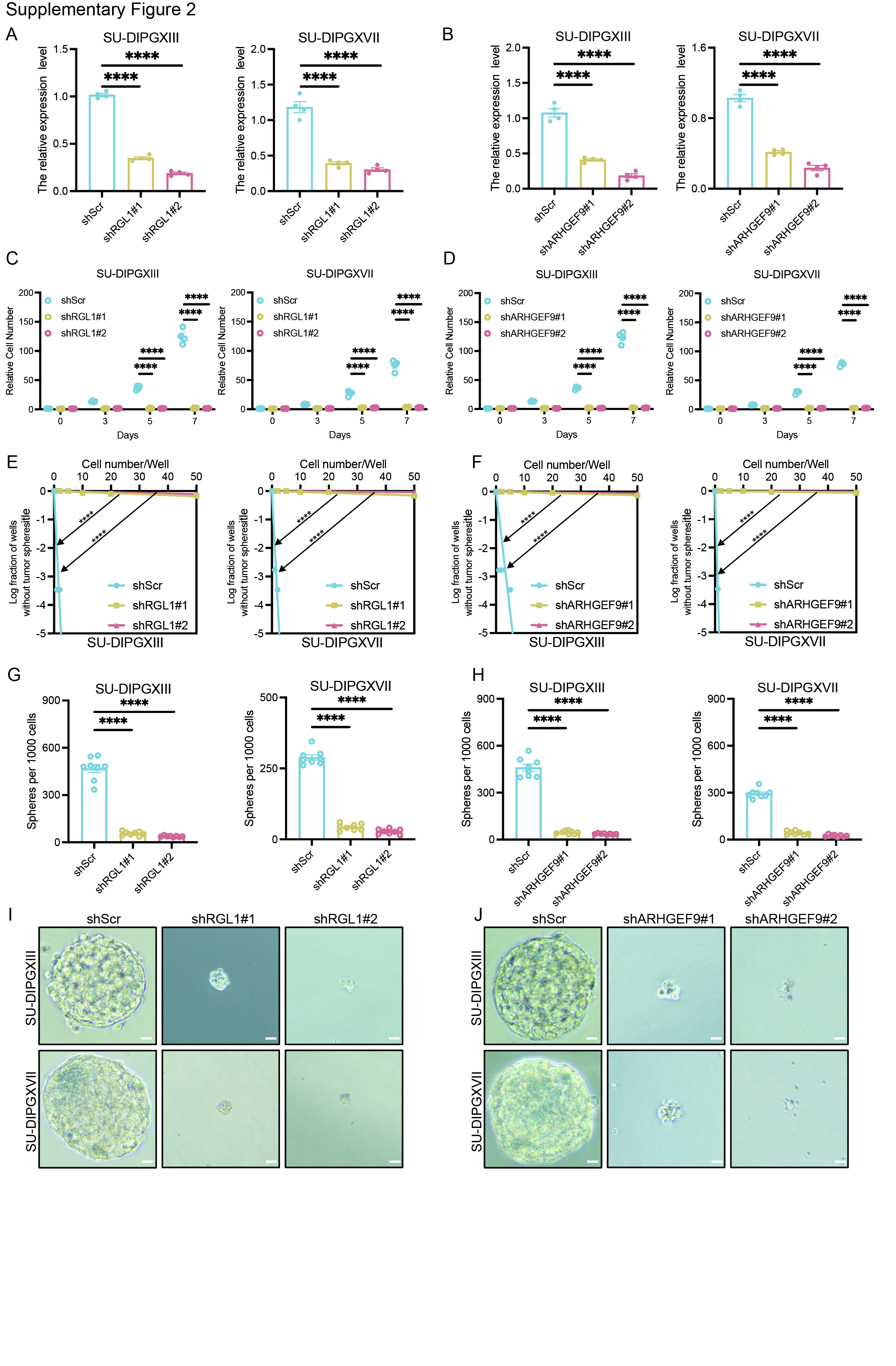

Supplement: Supplementary Figure 2 — RGL1 and ARHGEF9 targeting significantly inhibited tumor cell growth and stemness. (A) The quantification of RGL1 knockdown efficiency in SU-DIPGXIII (left) and SU-DIPGXVII (right). (B) The quantification of ARHGEF9 knockdown efficiency in SU-DIPGXIII (left) and SU-DIPGXVII (right). (C) The proliferation assay on cell number shows that RGL1 knockdown impairs the proliferation of SU-DIPGXIII (left) and SU-DIPGXVII (right). (D) The proliferation assay on cell number shows that ARHGEF9 knockdown impairs the proliferation of SU-DIPGXIII (left) and SU-DIPGXVII (right). (E) The limiting dilution assays in SU-DIPGXIII (left) and SU-DIPGXVII (right) cell lines with RGL1 knockdown. (F) The limiting dilution assays in SU-DIPGXIII (left) and SU-DIPGXVII (right) cell lines with ARHGEF9 knockdown. (G) The quantitative result of tumorsphere formation in vitro of RGL1 knockdown in SU-DIPGXIII (left) and SU-DIPGXVII (right). (H) The quantitative result of tumorsphere formation in vitro of ARHGEF9 knockdown in SU-DIPGXIII (left) and SU-DIPGXVII (right). (I) The result of tumorsphere formation in vitro shows impairing self-renewal of RGL1 knockdown in SU-DIPGXIII (left) and SU-DIPGXVII (right). (J) The result of tumorsphere formation (scale bar = 20 μm) in vitro shows impairing self-renewal of ARHGEF9 knockdown in SU-DIPGXIII (left) and SU-DIPGXVII (right). ****P<0.0001. [file Image2.tif]

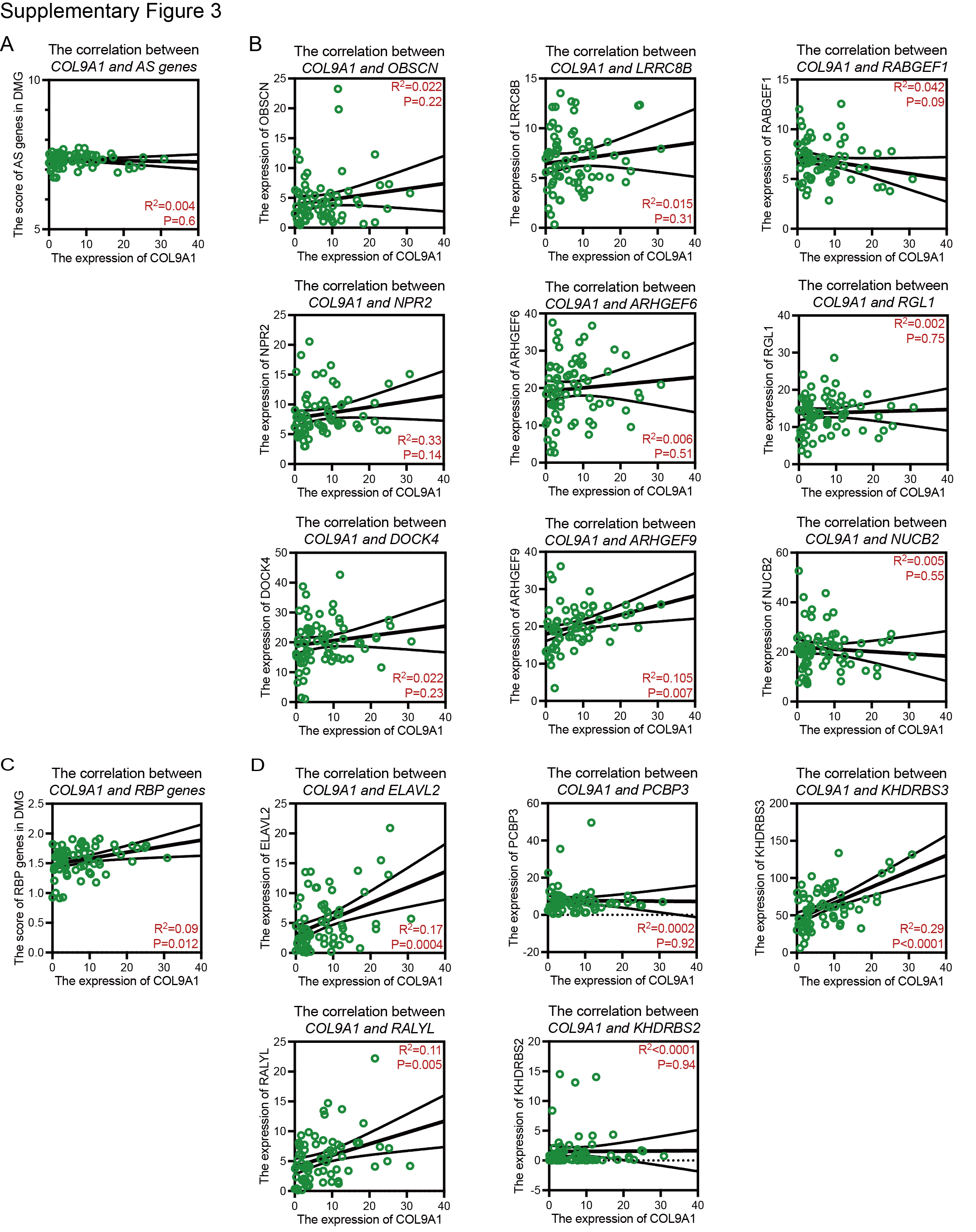

Supplement: Supplementary Figure 3 — The correlation analysis between the expression of COL9A1 and AS genes and RBP genes in DMG. (A) The correlation analysis between the expression of COL9A1 and AS genes set in DMG. (B) The correlation analysis between the expression of COL9A1 and each AS gene in DMG. (C) The correlation analysis between the expression of COL9A1 and RBP genes set in DMG. (D) The correlation analysis between the expression of COL9A1 and each RBP gene in DMG. [file Image3.tif]

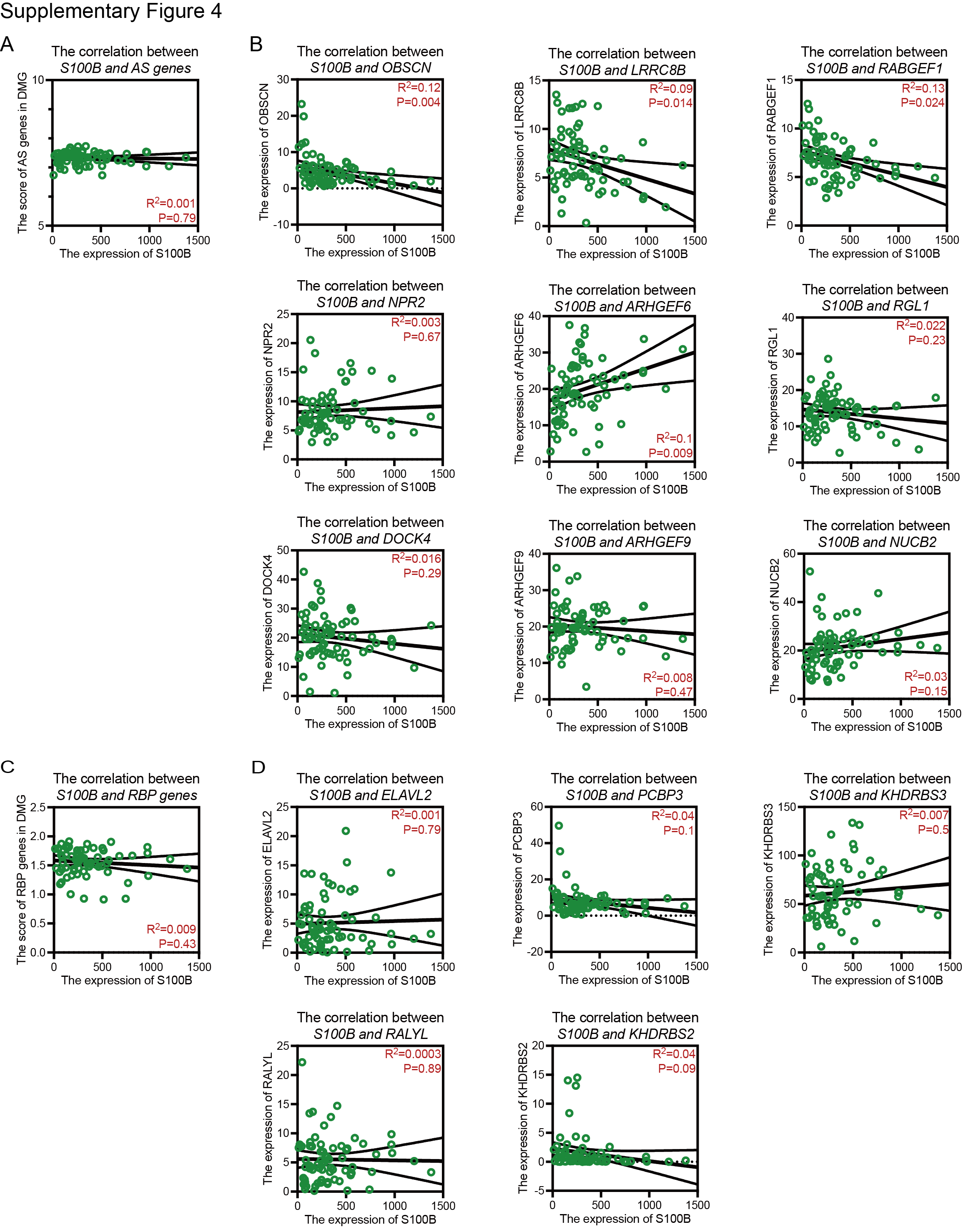

Supplement: Supplementary Figure 4 — The correlation analysis between the expression of S100B and AS genes and RBP genes in DMG. (A) The correlation analysis between the expression of S100B and AS genes set in DMG. (B) The correlation analysis between the expression of S100B and each AS gene in DMG. (C) The correlation analysis between the expression of S100B and RBP genes set in DMG. (D) The correlation analysis between the expression of S100B and each RBP gene in DMG. [file Image4.tif]

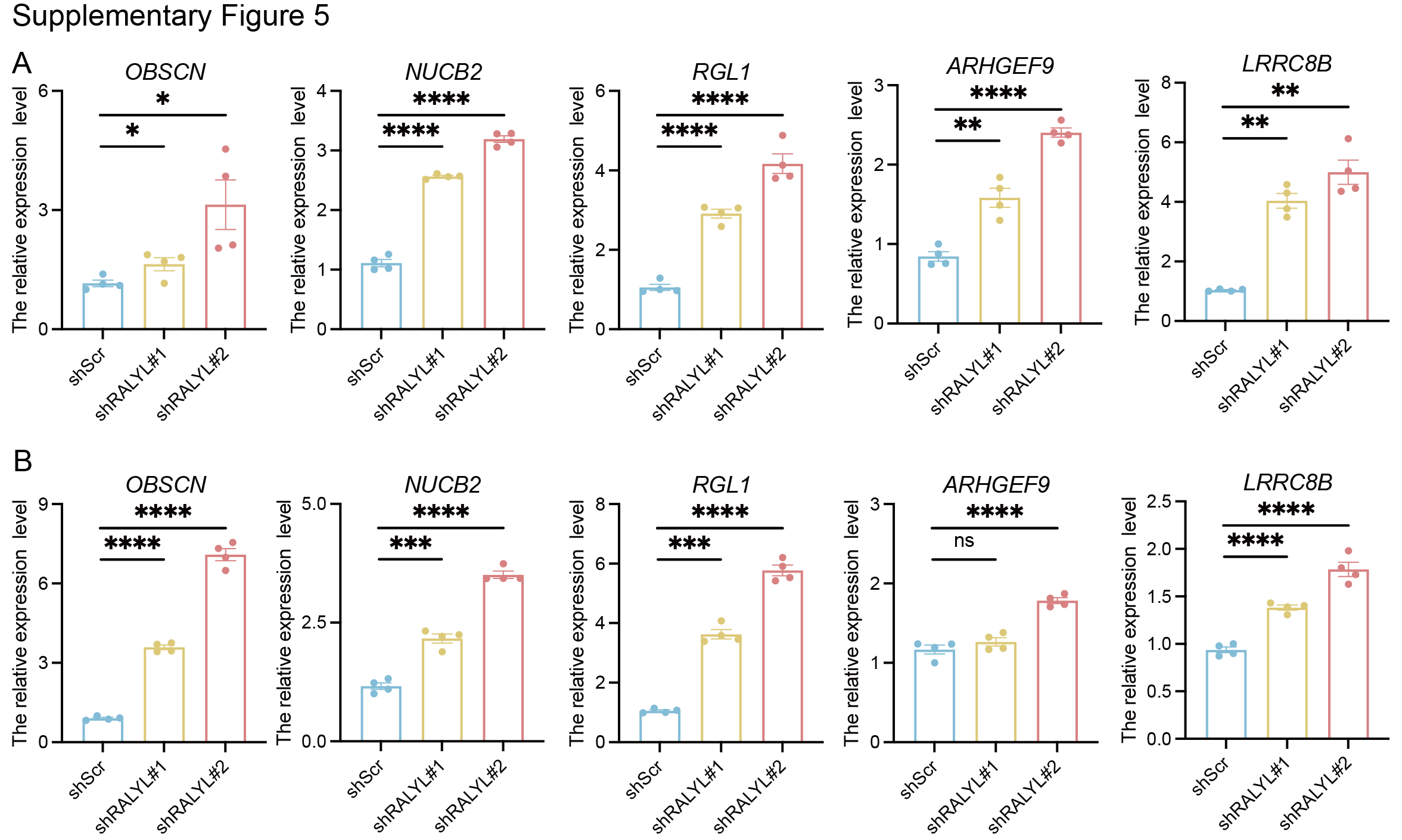

Supplement: Supplementary Figure 5 — The qRT-PCR validation of five AS genes in H3K27M DMG cell after RALYL knockdown. (A) The qRT-PCR validation of five AS genes in SU-DIPGXIII cell after RALYL knockdown. (B) The qRT-PCR validation of five AS genes in SU-DIPGXVII cell after RALYL knockdown. *P<0.05, ***P<0.001, ****P<0.0001, ns. P >0.05. [file Image5.tif]
